# Association of Burnout, Professional Fulfillment, and Self-care Practices of Physician Leaders With Their Independently Rated Leadership Effectiveness

**DOI:** 10.1001/jamanetworkopen.2020.7961

**Published:** 2020-06-16

**Authors:** Tait D. Shanafelt, Maryam S. Makowski, Hanhan Wang, Bryan Bohman, Mary Leonard, Robert A. Harrington, Lloyd Minor, Mickey Trockel

**Affiliations:** 1 Department of Internal Medicine, Stanford University School of Medicine, Stanford, California; 2 Stanford University School of Medicine, Stanford, California; 3 Department of Anesthesiology, Perioperative and Pain Medicine, Stanford University School of Medicine, Stanford, California; 4 Department of Pediatrics, Stanford University School of Medicine, Stanford, California; 5 Department of Otolaryngology, Stanford University School of Medicine, Stanford, California; 6 Department of Psychiatry and Behavioral Sciences, Stanford University School of Medicine, Stanford, California

## Abstract

**Importance:**

Although leadership behavior of physician supervisors is associated with the occupational well-being of the physicians they supervise, the factors associated with leadership behaviors are poorly understood.

**Objective:**

To evaluate the associations between burnout, professional fulfillment, and self-care practices of physician leaders and their independently assessed leadership behavior scores.

**Design, Setting, and Participants:**

This survey study of physicians and physician leaders at Stanford University School of Medicine (n = 1924) was conducted from April 1 to May 13, 2019. The survey included assessments of professional fulfillment, self-valuation, sleep-related impairment, and burnout. Physicians also rated the leadership behaviors of their immediate physician supervisors using a standardized assessment. Leaders’ personal well-being metrics were paired with their leadership behavior scores as rated by the physicians they supervised. All assessment scores were converted to a standardized scale (range, 0-10). Data were analyzed from October 20, 2019, to March 10, 2020.

**Main Outcomes and Measures:**

Association between leaders’ own well-being scores and their independently assessed leadership behavior.

**Results:**

Of 1924 physicians invited to participate, 1285 (66.8%) returned surveys, including 67 of 117 physician leaders (57.3%). Among these respondents, 651 (50.7%) were women and 729 (56.7%) were 40 years or older. Among the 67 leaders, 57 (85.1%) had their leadership behaviors evaluated by at least 5 physicians (median, 11 [interquartile range, 9-15]) they supervised. Overall, 9.8% of the variation in leaders’ aggregate leadership behavior scores was associated with their own degree of burnout. In models adjusted for age and sex, each 1-point increase in burnout score of the leaders was associated with a 0.19-point decrement in leadership behavior score (β = −0.19; 95% CI, −0.35 to −0.03; *P* = .02), whereas each 1-point increase in their professional fulfillment and self-valuation scores was associated with a 0.13-point (β = 0.13; 95% CI, 0.01-0.26; *P* = .03) and 0.15-point (β = 0.15; 95% CI, 0.02-0.29; *P* = .03) increase in leadership behavior score, respectively. Each 1-point increase in leaders’ sleep-related impairment was associated with a 0.15-point increment in sleep-related impairment among those they supervised (β = 0.15; 95% CI, 0.02-0.29; *P* = .03). The associations between leaders’ well-being scores in other dimensions and the corresponding well-being measures of those they supervised were not significant.

**Conclusions and Relevance:**

In this survey study, burnout, professional fulfillment, and self-care practices of physician leaders were associated with their independently assessed leadership effectiveness. Training, skill building, and support to improve leader well-being should be considered a dimension of leadership development rather than simply a dimension of self-care.

## Key Points

**Question:**

Are burnout, professional fulfillment, and self-care practices of physician leaders associated with their leadership effectiveness?

**Findings:**

In this survey study of 1285 physicians and physician leaders, each 1-point increase in the leaders’ burnout score was associated with a 0.19-point decrement in their independent leadership behavior score, whereas each 1-point increase in a leader’s professional fulfillment score was associated with a 0.13-point higher leadership behavior score. Overall, 9.8% of the variation in leaders’ aggregate leadership behavior scores was associated with a leader’s degree of burnout.

**Meaning:**

Burnout, professional fulfillment, and self-care practices of physician leaders appear to be associated with their leadership effectiveness.

## Teaser

This survey study assesses the association of burnout, professional fulfillment, and self-care practices of physician leaders with their independently assessed leadership behaviors.

## Introduction

These are challenging times for US medicine. Consolidation of medical practices, alternative payment models, new technologies, and a rapidly expanding medical knowledge base are transforming health care delivery. These forces have increased the complexity of care, created greater focus on productivity, heightened financial pressures, and altered the clinical encounter at the heart of physicians’ relationship with their patients.^1,2,3,4,5^

Work-related distress among physicians has also increased in association with these challenges and has implications for both physicians and patients.^6,7^ Most physicians now practice in organized groups, where effective leadership is important to address the continuously evolving challenges in the medical practice environment. Evidence indicates that the leadership behaviors of physician supervisors are strongly associated with professional fulfillment and burnout among the physicians they lead.^8^ This and similar findings on the importance of leadership to employee health^9,10^ indicate that greater attention to leader selection, development, evaluation, and feedback are an important component of organization-level efforts to reduce occupational distress among physicians and other health care professionals.^11^

Despite the association between leadership and occupational distress, little is known about how the well-being of physician leaders is associated with their leadership effectiveness. Although popular leadership books often discuss the importance of caring for self (eg, “sharpening the saw”),^12^^(p287)^ limited empirical data on how positive and negative dimensions of the leader’s own well-being affects their leadership performance are available. In addition, little is known about whether a leader’s own well-being and self-care behaviors are associated with the self-care and well-being habits of those they supervise (ie, well-being role modeling).^13^ In the present study, we evaluated burnout, professional fulfillment, and self-care indicators (eg, sleep health, self-valuation) of physician leaders and assessed the association of these results with independent evaluations of their leadership performance.

## Methods

### Participants and Survey Measures

Stanford University School of Medicine, Stanford, California, conducted a survey to inform organizational efforts to improve professional fulfillment and wellness among its physicians in the spring of 2019. All clinically credentialed Stanford faculty physicians (n = 1924) were invited to complete the electronic survey from April 1 to May 13, 2019. The survey was completed by practicing physicians as well as first-line physician leaders. All aspects of the study were reviewed by the Stanford University institutional review board and deemed exempt because they involved retrospective analysis of administratively collected data using a completely anonymized data set. Participation was voluntary, and the response rate of complete and partially completed surveys was determined using the American Association for Public Opinion Research (AAPOR) reporting guideline for cohort studies of internet surveys of named persons.^14^

### Evaluation of Professional Fulfillment, Burnout, Self-valuation, and Sleep-Related Impairment

The survey included standardized measures of positive and negative dimensions of well-being, including professional fulfillment, self-valuation,^15^ sleep-related impairment,^16^ and burnout.^17^ The measures and ratings are described below.

#### Burnout and Professional Fulfillment

The Professional Fulfillment Index was used to assess burnout and professional fulfillment.^17^ The Professional Fulfillment Index includes 4 items evaluating the work-exhaustion domain of burnout, 6 items assessing the interpersonal disengagement domain of burnout, and 6 items evaluating professional fulfillment. All burnout items are scored on a 5-point Likert scale with options ranging from “not at all” to “extremely” for burnout items and “not at all true” to “completely true” for professional fulfillment items. Aggregate scores for burnout and professional fulfillment were calculated using the published approach, with possible scores ranging from 0 to 40 and 0 to 24, respectively.^17^ The validity, sensitivity, and reliability of the Professional Fulfillment Index have been established.^15,17^

#### Sleep-Related Impairment

The National Institutes of Health Patient-Reported Outcomes Measurement Information System short-form version 1.0 Sleep-Related Impairment Scale, a validated assessment tool,^18^ was used to assess sleep-related impairment.^16^ This instrument includes 8 items evaluating symptoms of sleep-related impairment that are answered on 5-point Likert scales evaluating intensity ranging from “not at all” to “very much.” Aggregate scores were determined using the published approach (possible range, 8-40).^18^

#### Self-valuation

Self-valuation consists of a growth mindset (responding to errors and personal imperfection with a desire to learn and improve rather than shame) in combination with the ability to prioritize self-care and personal well-being. Self-valuation was assessed using the 4-item Clinician Self-valuation Scale.^15^ Responders indicate their experience with indicators of poor self-valuation in the past 2 weeks using a 5-point Likert scale (0 indicated always; 4, never). Using the standard scoring approach, a total self-valuation score ranging from 0 to 16 is calculated by summing the score of the individual items (with higher scores indicating more favorable self-valuation).

### Leadership Behaviors

All participants were asked to select the name of their immediate supervisor from a drop-down menu that listed the names of their department chair, division chiefs, and medical clinic directors. They then evaluated this leader using the Mayo Clinic Participatory Management Leadership Index.^8^ This instrument was designed to evaluate leadership behaviors associated with team member engagement, including dimensions related to inclusion (treating everyone with respect), keeping people informed, soliciting input, empowering team members, nurturing professional development, and providing feedback and recognition. The original 12-item index was subsequently revised to a 9-item instrument (eAppendix in the Supplement). Each item is scored on a 5-point scale (1 indicates strongly disagree; 5, strongly agree), and the scores from the individual items are summed to yield a total score (possible range, 9-45, with higher scores indicating more favorable leadership behavior). The aggregate leader behavior score for each leader evaluated was determined by the composite evaluations of all responding physicians who they supervised.

### Pairing of Leader and Physician Data Sets

To preserve confidentiality, an independent, institutionally approved third party administrator without access to personnel or other employment records paired scores on leaders’ occupational burnout, professional fulfillment, and self-care practices (eg, sleep-related impairment, self-valuation) with their leadership behavior scores as assessed by the physicians they supervised. The third-party administrator also paired occupational well-being and self-care indicator scores of these physicians with the same well-being and self-care indicators of their leaders. The administrator then removed all identifiers from the anonymized paired data sets before sending them to the study biostatistician (H.W.) for analysis. The anonymized data set was constructed to ensure that the study statistician did not have the ability to identify any specific leader, individual responder, or division/work unit through their analysis. Leaders evaluated by at least 5 of their physician reports were included in analyses.

### Statistical Analysis

Data were analyzed from October 20, 2019, to March 10, 2020. All analyses were conducted in R, version 3.6.0 (R Core Team, 2019), with all *P* values specified as 2 sided and results deemed statistically significant at *P* < .05. Standard descriptive summary statistics were used to characterize the physician and leader samples. All instruments were scored using the standard, published approach followed by normalization of each scale to a 0- to 10-point scale for simple interpretation of results. Differences in demographic characteristics, burnout, professional fulfillment, and self-care practices of leaders and the physicians they supervised were compared using the Welch *t* test for continuous features and χ^2^ test for categorical features. Mixed-effects models—to account for the nested data structure of multiple physicians reporting to individual leaders—were specified to test the associations between burnout, professional fulfillment, and self-care indicators of physician leaders and their independently rated leadership behaviors, with and without adjustment for sex and age. Mixed-effects models were also specified to test the associations among leaders’ burnout, professional fulfillment, and self-care indicators on the corresponding variables observed in those they supervised with and without adjustments for sex and age. Ordinary least squares regression was used to estimate the portion of variance in the leaders’ mean leadership behavior rating attributable to their occupational burnout and professional fulfillment.

## Results

Of the 1924 physicians invited to participate, 1285 (response rate, 66.8%) returned surveys. Among these, 651 (50.7%) were women and 634 (49.3%) were men; 729 (56.7%) were 40 years or older. Among the 117 physician leaders evaluated, 67 (57.2%) personally completed their own wellness survey. Among these 67 leaders, 57 (85.1%) had their leadership behavior independently evaluated by at least 5 physicians from their unit and were included in analyses. A total of 813 leadership evaluations for these 57 first-line leaders were received (median, 11 [interquartile range, 9-15] evaluations per leader).

The demographic characteristics and the burnout, professional fulfillment, and self-care scores of these 57 physician leaders and the 820 physicians they supervised are shown in the Table. Physician leaders were older than physicians who were not in a leadership role (aged 30-39 years, 0 leaders vs 239 of 721 nonleaders [33.1%]) and were more likely to be men (38 of 54 leaders [70.4%] vs 371 of 796 nonleaders [46.6%]; *P* = .001). Leaders worked 14 hours more each week than physicians who were not in a leadership role (mean [SD], 68.4 [11.8] vs 54.5 [15.4] h/wk; *P* < .001). The mean (SD) burnout score of physician leaders was lower than that of physicians not in a leadership role (2.14 [2.00] vs 2.82 [1.86]; *P* = .02) whereas the mean (SD) professional fulfillment score was higher (7.44 [2.38] vs 6.51 [1.99]; *P* = .005). Although the difference in professional fulfillment scores persisted after adjusting for age and sex, the difference in burnout scores between physician leaders and physicians who were not in leadership positions was not significant after adjusting for age and sex. No significant differences in self-valuation or sleep-related impairment scores were observed between physician leaders and physicians who were not in a leadership role. Leadership behavior scores for physician leaders ranged from 0 to 10 with a mean (SD) score of 7.59 (2.32). No difference in leadership behavior score was observed by leader age, sex, or work hours (eTable in the Supplement).

**Table.** Demographic Characteristics of Physicians and Leaders

Variables	Participant group^a^			*P* value^b^
	All physicians (n = 877)	Leaders (n = 57)	Nonleader physicians (n = 820)	
Age group, y				
30-39	239/768 (31.1)	0	239/721 (33.1)	<.001
40-49	219/768 (28.5)	5/47 (10.6)	214/721 (29.7)	
50-59	163/768 (21.2)	23/47 (48.9)	140/721 (19.4)	
≥60	147/768 (19.1)	19/47 (40.4)	128/721 (17.8)	
Missing	109/877 (12.4)	10/57 (17.5)	99/820 (12.1)	.32
Sex				
Male	409/850 (48.1)	38/54 (70.4)	371/796 (46.6)	.001
Female	441/850 (51.9)	16/54 (29.6)	425/796 (53.4)	
Missing	27/877 (3.1)	3/57 (5.3)	24/820 (2.9)	.56
Work hours per week				
Mean (SD)	55.34 (15.55)	68.4 (11.8)	54.5 (15.4)	<.001
Missing	2/877 (0.2)	1/57 (1.8)	1/820 (0.1)	.13
Clinician Self-valuation Scale score^c^				
Mean (SD)	4.84 (2.19)	4.39 (2.28)	4.87 (2.18)	.12
Missing	1/877 (0.1)	0	1/820 (0.1)	>.99
Sleep-Related Impairment Scale score^d^				
Mean (SD)	3.28 (1.75)	2.86 (1.68)	3.31 (1.76)	.06
Missing	1/877 (0.1)	0	1/820 (0.1)	>.99
Burnout score^e^				
Mean (SD)	2.78 (1.88)	2.14 (2.00)	2.82 (1.86)	.02
Missing	3/877 (0.3)	0	3/820 (0.4)	>.99
Professional Fulfillment Index score^f^				
Mean (SD)	6.57 (2.03)	7.44 (2.38)	6.51 (1.99)	.005
Mayo Clinic Participatory Management Leadership Index score^g^				
Mean (SD)	NA	7.59 (2.32)	NA	NA
Missing	NA	0	NA	NA

Abbreviation: NA, not applicable.

^a^ Unless otherwise indicated, data are expressed as number/total number (percentage) of physicians with available data. Percentages have been rounded and may not total 100.

^b^ Calculated using Welsh *t* test for continuous variable and χ^2^ test for categorical variables.

^c^ Scores range from 0 to 16, with higher scores indicating more favorable self-valuation.

^d^ Scores range from 0 to 10, with higher scores indicating greater impairment.

^e^ Scores range from 0 to 10, with higher scores indicating greater burnout.

^f^ Scores range from 0 to 10, with higher scores indicating greater professional fulfillment.

^g^ Independently rated by the physicians that leader supervised. Scores range from 0 to 10, with higher scores indicating more favorable leadership behavior.

### Association of Leaders’ Wellness Scores With Independent Assessment of Leadership Behaviors 

**Figure 1.**
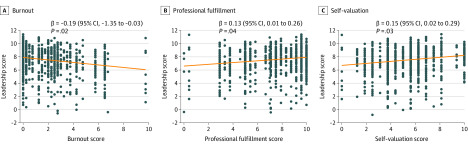
Partial Residual Plots of Leaders’ Personal Burnout, Professional Fulfillment, and Self-valuation Associated With Their Independently Rated Leadership Effectiveness Data are adjusted for leader sex and age.

The overall level of burnout, professional fulfillment, and self-valuation of physician leaders were associated with their leadership behavior score as independently rated by the physicians they supervised. Overall, 9.8% of the variation in leaders’ aggregate leadership behavior score—determined by the mean of the evaluations received from those they lead—was associated with their own degree of burnout in the bivariate model. Each 1-point increase in a leader’s own burnout score was associated with a 0.15-point decrement in their leadership behavior score (95% CI, −0.29 to −0.01; *P* = .03). This finding persisted after adjusting for leader sex and age (β = −0.19; 95% CI, −0.35 to −0.03; *P* = .02) (Figure 1A).

Overall, 7.9% of the variation in leaders’ aggregate leadership behavior score was associated with their own degree of professional fulfillment in a bivariate model. Each 1-point increase in professional fulfillment scale was associated with a 0.12-point higher leadership behavior score (95% CI, 0.002-0.24; *P* = .047). This association persisted after adjusting for sex and age (β = 0.13; 95% CI, 0.01-0.26; *P* = .04) (Figure 1B).

Overall, 8.5% of the variation in leaders’ aggregate leadership behavior score was associated with their own degree of self-valuation in a bivariate mode. Each 1-point increase in a leader’s self-valuation score scale was associated with a 0.14-point improvement in their leadership behavior score (95% CI, 0.01-0.26; *P* = .03), a finding that again persisted after adjusting for sex and age (β = 0.15; 95% CI, 0.02-0.29; *P* = .03) (Figure 1C). There was no association between leaders’ sleep-related impairment and ratings of their leadership behavior before (β = −0.09; 95% CI, −0.23 to 0.05; *P* = .20) or after (β = −0.11; 95% CI, −0.26 to 0.04; *P* = .14) adjusting for leader sex and age.

### Association Between Leader and Physician Wellness Scores

To evaluate well-being role modeling by leaders, we next evaluated whether physicians’ own burnout, professional fulfillment, and self-care scores were associated with the same domains in their leaders. Physicians’ level of burnout was not significantly associated with their leaders’ level of burnout before (β = 0.07; 95% CI, −0.02 to 0.15; *P* = .13) or after (β = 0.03; 95% CI, −0.05 to 0.11; *P* = .43) adjusting for sex and age. Physicians’ professional fulfillment was associated with the professional fulfillment of their leaders before (β = 0.08; 95% CI, 0.01-0.16; *P* = .03) but not after (β = 0.06; 95% CI, −0.02 to 0.13; *P* = .13) adjusting for sex and age. Physicians’ self-valuation was not associated with the self-valuation of their leaders before (β = 0.02; 95% CI, −0.06 to 0.11; *P* = .56) or after (β = 0.01; 95% CI, −0.08 to 0.07; *P* = .85) adjusting for sex and age. Each 1-point increase in leaders’ sleep-related impairment score was associated with a 0.18-point increment in the sleep-related impairment score of physicians they supervised (95% CI, 0.09-0.27; *P* < .001), an association that persisted after adjusting for sex and age (β = 0.15; 95% CI, 0.06-0.24; *P* = .001).

## Discussion

We report herein the first study, to our knowledge, that evaluates the association between leaders’ own level of well-being and their independently rated leadership behavior. Significant associations were observed between leaders’ level of burnout as well as professional fulfillment and their leadership behaviors as assessed by the physicians they led, findings that persisted after adjusting for leader sex and age. A significant association between leaders’ own level of self-valuation and their leadership behavior score was also observed. Approximately 10% of the variation in aggregate, independently rated leadership behavior scores was associated with leaders’ own burnout scores in bivariate analysis.

**Figure 2.**
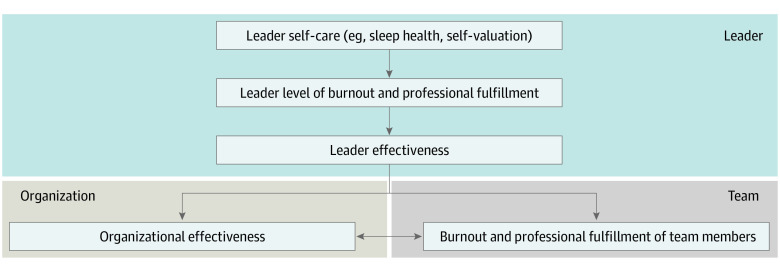
Leader Well-being and Effectiveness This proposed model shows how the burnout, professional fulfillment, and self-care of physician leaders may affect the organization and the well-being of those physicians they lead.

These results indicate that leaders’ own levels of burnout, professional fulfillment, and self-valuation are associated with their leadership behavior. Previous studies have found that the leadership behavior score of physician leaders is strongly associated with the levels of burnout and professional satisfaction among the physicians in their work units.^8^ Collectively, these studies provide evidence that a leader’s occupational well-being primarily affects the well-being of their team indirectly by eroding leader effectiveness (Figure 2). These findings have critical implications for organizational efforts to enhance leadership effectiveness and reduce occupational distress among physicians and other health care professionals. Specifically, they would argue for prioritizing leader well-being and inclusion of training, skill building, and other support to foster leaders’ well-being as part of leadership development efforts (as opposed to simply as self-care).^19^

The amount of variation in leadership effectiveness associated with leaders’ own well-being is noteworthy. The results suggest that more than 90% of a leader’s effectiveness is explained by other variables, presumably their aptitude and skill in various leadership domains such as participatory management, relationship building, ability to foster trust, communication, inclusion, human development, aligning values, and team building^20,21,22^ (in addition to rater factors attributable to the cohorts of physicians who rate them). They also suggest that, for leaders with similar aptitude in such domains, as much as 10% of their leadership effectiveness is associated with their own well-being (burnout, professional fulfillment, and self-valuation). Accordingly, although the training and skill building to advance a portion of the residual 90% of leader effectiveness may vary greatly by leader and role, we identify herein a dimension of leadership development that may have near universal applicability to all leaders, a premise that would suggest it receive meaningful attention as a foundational component of organizational leadership development efforts.

We believe framing attention to well-being in these domains as a leadership attribute, rather than a self-care issue, is also more likely to alter leaders’ behavior. Although the leadership literature has long suggested that leaders should attend to their own health to be effective in their leadership roles,^12^ the lack of empirical evidence to substantiate these suggestions likely reduces prioritization of this domain by organizations and leaders. In medicine, physicians subjugate their own needs for the needs of patients on a regular basis, even when this is detrimental to their own well-being.^15^ One of the most powerful motivators for change to improve self-care behaviors among physicians is the belief that it is beneficial for their patients.^23,24,25,26,27^ In analogous manner, evidence that leaders should attend to their own well-being to improve their leadership effectiveness may increase the proportion of leaders who engage in such behavior.

The link between a leader’s own well-being and leadership behavior may be mediated, in part, through emotional intelligence—the ability to understand and manage one’s own emotions, empathize with others, and manage relationships effectively.^21,28^ Previous studies have demonstrated an association between self-valuation and emotional intelligence, suggesting a potential mechanism through which self-care may influence leaders’ efficacy.^29,30^ One study among fire fighters also found that supervisor scores on self-kindness, a similar concept to self-valuation, were positively correlated with crew-member ratings of their supervisor.^31^

We did not observe a significant association between leaders’ own level of burnout, professional fulfillment, or self-valuation and the scores of the physicians they led in these same domains. In contrast, an association between leaders’ sleep-related impairment and sleep-related impairment scores among those they supervised was observed. Although this observation may be related to shared specialty (ie, the schedules and work hours of some specialties are more likely to interfere with sleep), this observation is consistent with previous observations outside medicine. These prior studies suggest that when leaders publicly devalue sleep (such as boasting that they are productive because of how little they sleep^32^), the members of their team sleep a mean of 25 minutes less each night.^33^ Previous studies have also found a link between a leader’s sleep health and leadership effectiveness through an effect on the leader’s relationships with team members.^33,34,35,36^

### Limitations

Our study is subject to several limitations. First, although the participation rate was high and standardized instruments were used to evaluate all dimensions assessed, the results are derived from a single institution, and the overall number of leaders evaluated was modest. Second, although all leaders had a minimum of 5 leader evaluations, the number of evaluations per leader was, in some cases, limited. Although this aspect is inherent given our focus on first-line leaders (who lead smaller teams), it is a limitation nonetheless. Third, although we evaluated multiple dimensions of well-being (burnout, professional fulfillment) and self-care (sleep, self-valuation), other domains of self-care, such as exercise, nutrition, stress reduction, and work-life integration habits were not assessed. Finally, although the assumption that leader well-being supports leadership effectiveness is consistent with theoretical constructs, the interactions between these dimensions are likely complex, multifaceted, and potentially bidirectional. For example, leaders who sense that physicians who report to them do not value their leadership (eg, lower leader behavior score) may have lower professional fulfillment and be at higher risk for burnout.

## Conclusions

We report empirical evidence that leaders’ own level of burnout, professional fulfillment, and self-valuation were associated with their independently assessed leadership behavior. A leader’s personal behavior with respect to sleep health also may have an important role-modeling effect on those they lead. These findings provide important proof-of-concept insights into how leaders’ own well-being might affect their leadership effectiveness and the well-being of those they lead. The results indicate that organizations should prioritize the well-being of leaders as an important driver of leader effectiveness and provide training, skill building, and additional support to improve leader well-being as an integral element of leadership development efforts. Given the large association of leadership behavior with burnout and professional fulfillment among team members,^8^ prioritizing leader well-being may be an important strategy to promote the well-being of all individuals in the organization.

## Notes

Supplement. **eAppendix.** Mayo Clinic Participatory Management Leadership Index **eTable.** Leaders’ Personal Characteristics and Leadership Behavior Score
