# Error in Data in Abstract, Methods, and Results

**DOI:** 10.1001/jamanetworkopen.2020.21812

**Published:** 2020-09-11

In the Original Investigation titled “Association of Burnout, Professional Fulfillment, and Self-care Practices of Physician Leaders With Their Independently Rated Leadership Effectiveness,”^1^ published online June 16, 2020, some data were transposed and a sample description was missing in the Abstract, Methods, and Results. This article has been corrected.^1^
