# Supplementary material for: Association of Burnout, Professional Fulfillment, and Self-care Practices of Physician Leaders With Their Independently Rated Leadership Effectiveness
Source: JAMA Netw Open. 2020 Jun 16;3(6):e207961. doi: 10.1001/jamanetworkopen.2020.7961 (PMC7298612; doi:10.1001/jamanetworkopen.2020.7961)
Supplement: Supplement. — eAppendix. Mayo Clinic Participatory Management Leadership Index eTable. Leaders’ Personal Characteristics and Leadership Behavior Score [file jamanetwopen-3-e207961-s001.pdf]

Supplementary Online Content

Shanafelt TD, Makowski MS, Wang H, et al. Association of burnout, professional fulfillment, and self-care practices of physician leaders with their independently rated leadership effectiveness. *JAMA Netw Open*. 2020;3(6):e207961. doi:10.1001/jamanetworkopen.2020.7961

eAppendix. Mayo Clinic Participatory Management Leadership Index
eTable. Leaders' Personal Characteristics and Leadership Behavior Score

This supplementary material has been provided by the authors to give readers additional information about their work.

©2020 Shanafelt TD et al. *JAMA Network Open*.

eAppendix. Mayo Clinic Participatory Management Leadership Index

To what extent do you agree or disagree with each of the following statements about (name of immediate supervisor)*?*
Holds career development conversations with me[1]
Empowers me to do my job[1]
Encourages employees to suggest ideas for improvement[1]
Treats me with respect and dignity[1]
Provides helpful feedback and coaching on my performance[1]
Recognizes me for a job well done[1]
Keeps me informed about changes taking place at **Name of organization**[1]
Encourages me to develop my talents and skills[1]
Overall, how satisfied are you with (name of immediate supervisor)[2]

[1] response options: 5 =strongly agree, 4=agree, 3=neither agree nor disagree, 2=disagree, 1=strongly disagree; NA=don't know/not applicable

[2] response options: 5=very satisfied, 4=satisfied, 3=neither satisfied nor dissatisfied, 2=dissatisfied, 1=very dissatisfied

Scoring: sum 1-5 score for each of 9 items to generate a total score (range 9-45). Total scores can be normalized to a 0-10 scale by calculating the mean 1-5 score for the leadership items answered (for those answering at least 7 items), subtracting 1, and multiplying by 2.5.

Used with permission, copyright Mayo Clinic.

eTable. Leaders' Personal Characteristics and Leadership Behavior Score

Characteristics	Leader Behavior Score[1] (Mean +/- SD)	P-Value
Leader Gender		
Men (N=38)	7.79 +/- 0.94	0.677
Women (N=16)	7.63 +/- 1.35	
Leader Age		
40-49 (N=5)	7.96 +/- 1	0.879
50-59 (N=23)	7.8 +/- 0.92	
≥60 (N=19)	7.73 +/- 0.96	
Leader Work hours		
Q1: <60 (N=19)	7.71 +/- 0.94	0.927
Q2: 60-68 (N=10)	7.94 +/- 0.85	
Q3: 68-75 (N=15)	7.65 +/- 1.07	
Q4: 75-120 (N=12)	7.71 +/- 1.49	

[1] Independently rated by the physicians the leader supervised

©2020 Shanafelt TD et al. *JAMA Network Open*.
